# Microbial contamination of mobile phones in a health care setting in Alexandria, Egypt

**DOI:** 10.3205/dgkh000246

**Published:** 2015-02-02

**Authors:** Heba Sayed Selim, Amani Farouk Abaza

**Affiliations:** 1 Microbiology Department, High Institute of Public Health, Alexandria University, Alexandria, Egypt

**Keywords:** mobile phones, bacterial contamination, hand hygiene, MRSA, ESBL

## Abstract

**Aim:** This study aimed at investigating the microbial contamination of mobile phones in a hospital setting.

**Methods:** Swab samples were collected from 40 mobile phones of patients and health care workers at the Alexandria University Students’ Hospital. They were tested for their bacterial contamination at the microbiology laboratory of the High Institute of Public Health. Quantification of bacteria was performed using both surface spread and pour plate methods. Isolated bacterial agents were identified using standard microbiological methods. Methicillin-resistant *Staphylococcus aureus* was identified by disk diffusion method described by Bauer and Kirby. Isolated Gram-negative bacilli were tested for being extended spectrum beta lactamase producers using the double disk diffusion method according to the Clinical and Laboratory Standards Institute recommendations.

**Results:** All of the tested mobile phones (100%) were contaminated with either single or mixed bacterial agents. The most prevalent bacterial contaminants were methicillin-resistant *S. aureus* and coagulase-negative staphylococci representing 53% and 50%, respectively. The mean bacterial count was 357 CFU/ml, while the median was 13 CFU/ml using the pour plate method. The corresponding figures were 2,192 and 1,720 organisms/phone using the surface spread method.

**Conclusions:** Mobile phones usage in hospital settings poses a risk of transmission of a variety of bacterial agents including multidrug-resistant pathogens as methicillin-resistant *S. aureus*. The surface spread method is an easy and useful tool for detection and estimation of bacterial contamination of mobile phones.

## Zusammenfassung

**Zielsetzung:** Es sollte die mikrobielle Kontamination von Mobiltelefonen in einer universitären Gesundheitseinrichtung untersucht werden.

**Methode:** Abstrichproben wurden von 40 Mobiltelefonen von Patienten und Mitarbeitern im Universitätskrankenhaus in Alexandria entnommen. Die mikrobiologische Analyse wurde im mikrobiologischen Labor des Instituts für Public Health durchgeführt. Die Quantifizierung erfolgte sowohl durch direkte Ausbringung auf die Platte als auch durch Anlage von Subkulturen zur Differenzierung. Methicillin-resistente *Staphylococcus aureus* (MRSA) wurden mittels Plättchendiffusionsmethode nach Bauer und Kirby identifiziert. Isolierte Gram-negative Organismen wurden auf Vorkommen von ESBL-Bildung mittels der Doppeldiffusionsmethode gemäß Empfehlung des Clinical and Laboratory Standards Institute untersucht.

**Ergebnisse:** Alle untersuchten Mobiltelefone waren entweder mit einer oder mehreren bakteriellen Arten kontaminiert. Am häufigsten wurden MRSA (53%) und Koagulase-negative Staphylokokken (50%) nachgewiesen. Als mittlere Anzahl von KbE ergaben sich 357 KbE mit einem Median von130 KbE/ml pro Mobiltelefon im Plattengussverfahren. Die korrespondierenden Werte betrugen 2.192 bzw. 1.720 KbE/Mobiltelefon im Direktausstrich.

**Schlussfolgerung:** Mobiltelefone stellen ein Risiko in Gesundheitseinrichtungen zur Weiterverbreitung nosokomialer Pathogen einschließlich MRSA dar. Auf der Oberfläche von Mobiltelefonen kann die mikrobielle Kontamination methodisch einfach nachgewiesen werden.

## Introduction

Mobile phones have become one of the most indispensable accessories of professional and social life. They are increasingly becoming an important means of communication worldwide being easily accessible, economical and user-friendly. They are widely used by the healthcare workers (HCWs) and non-HCWs equally in every location. With all the achievements and benefits of the mobile phone, it is easy to overlook the health hazard it might pose to its many users [1]. 

The constant handling of mobile phones by users in hospitals (by patients, visitors and HCWs, etc.) makes it an open breeding place for transmission of microorganisms, as well as health care-associated infections (HAIs). This is especially so with those associated with the skin due to the moisture and optimum temperature of human body especially our palms [2]. These factors and the heat generated by mobile phones contribute to harboring bacteria on the device at alarming levels. When we consider a phone's daily contact with the face, mouth, ears, and hands, the dire health risks of using germ-infested mobile devices are obvious [3]. 

Unlike our hands, which are easily disinfected using alcohol-based hand rubs (ABHRs) that are made available readily across all hospitals and medical facilities, our mobile phones are cumbersome to clean. We even rarely make an effort to disinfect them. As a result, these devices have the potential for contamination with various bacterial agents [4]. 

Doctors and healthcare staff working in critical areas as intensive care units (ICUs) and operating units are highly exposed to deadly micro-organisms. These mobile phones used by HCWs often become carriers and may serve as vectors and spread microorganisms wherever they are taken along [5]. Colonized micro-organisms on the devices of HCWs may be transmitted to patients even if patients do not have direct contact with mobile phones [6]. These organisms if pathogenic can be detrimental to the health of the patients especially those in critical care units and if the organisms transferred happen to be drug-resistant; the situation becomes even more grave as it becomes difficult to treat because of the limited drug options available [7]. 

HAIs affect more than 25 percent of admitted patients in developing countries. In U.S. hospitals, they cause 1.7 million infections per year and are associated with approximately 100,000 deaths. It is estimated that one third of these infections could be prevented by adhering to standard infection control guidelines [8]. Multidrug-resistant (MDR) bacteria are commonly implicated in HAIs and can be challenging to eliminate [9].

This study was conducted to investigate bacterial contamination of mobile phones in a hospital setting.

## Materials and methods

### Study design, sample size and study setting

This cross sectional study was carried out during a period of a month and a half from the beginning of March 2014 till the middle of April 2014. 

Using Epiinfo version 6 based on the desire of detecting a prevalence of mobile phone contamination of 97.5% [10] and using a 95% confidence level and a 5% error around the expected prevalence and an alpha error of 5%, the resulting minimum sample size required amounted to 38 mobile phones. 

A total of 40 mobile phones of patients and HCWs at the Alexandria University Students' Hospital (AUSH) were tested for their bacterial contamination. The examined mobile phones were randomly collected from 4 departments: laboratory, ICU, dialysis unit and triage area (10 mobile phones from each department). A questionnaire was used for data collection of all the relevant information on tested mobile phones. Oral consents were obtained from all individuals whose mobile phones were included in the present study.

### Samples collection and processing

Samples from mobile phones were collected using sterile cotton swabs. Each swab was first moistened with sterile peptone water and was rotated over the surface of both sides of the tested mobile phone together with the keypad in non touchscreen phones. All swabs were immediately streaked (surface spread) over the surface of blood and MacConkey’s agar plates. The cotton ends of these swabs were cut off and soaked in 10 ml peptone water. All inoculated blood and MacConkey’s agar plates together with the inoculated peptone water tubes were transferred rapidly to the microbiology laboratory at the High Institute of Public Health (HIPH). 

At the laboratory, blood and MacConkey’s agar plates were incubated aerobically at 37°C for 24 hours. The inoculated peptone water tubes were vortexed and a one ml from each tube was placed in a sterile petridish, then 15 ml of melted plate count agar medium was poured over the sample portion. The agar was thoroughly mixed with the sample portion and allowed to set and solidify. The plates were then inverted and incubated aerobically at 37oC for 24 hours. 

#### Quantification of bacterial isolates 

The number of estimated colony forming units (CFU) for each sample subjected to pour plate (PP) method was then counted using the Quebec colony counter (Reichert, USA) and recorded as CFU/ml. Isolated colonies on blood and MacConkey’s agar plates using surface spread (SS) method were counted and recorded as organisms/phone. 

#### Identification of isolates

Isolated bacterial agents were identified according to the standard microbiological methods described by Forbes et al. (2007) [11]. They were identified using Gram’s staining, colony morphology and appropriate biochemical tests. For identification of Gram-positive cocci (GPC); isolates that appeared as medium sized circular, white or golden yellow with smooth convex surface and entire edge and were β-hemolytic or non-hemolytic on blood agar plates and were positive for catalase, slide and tube coagulase and Voges Proskauer tests were considered as *Staphylococcus aureus* (*S. aureus*). Non-haemolytic, catalase-positive, coagulase-negative, bacitracin-sensitive GPC were identified as *Micrococcus* spp., while catalase-positive, coagulase-negative and bacitracin-resistant GPC were considered as coagulase-negative staphylococci (CoNS). 

*S. aureus* and CoNS identified isolates were further checked for their susceptibility to methicillin using oxacillin (1 µg) and cefoxitin (30 µg) discs on Mueller Hinton agar plates supplemented by 4% NaCl by disk diffusion method described by Bauer and Kirby [12]. The inhibition zone diameters were measured and interpreted as recommended by the Clinical and Laboratory Standards Institute (CLSI) [13]. 

As regards Gram-negative bacilli (lactose and non-lactose fermenters), the oxidase, catalase, triple sugar iron agar (TSI), indole, methyl red, Voges Proskauer, citrate (IMViC) and urease tests were carried out for their identification. They were further tested for being extended spectrum beta-lactamase (ESBL) producers using the double disk diffusion method according to CLSI recommendations. Ceftazidime 30 µg, ceftazidime-clavulanate 30/10 µg, cefotaxime 30 µg and cefotaxime-clavulanate 30/10 µg discs were used. A ≥5 mm increase in a zone diameter for either antimicrobial agent tested in combination with clavulanate vs. the zone diameter of the agent when tested alone confirmed ESBL producers [13]. 

## Statistical analysis

Data were analyzed [14] using SPSS version 16.0, the 0.05 level was used as the cut off value for statistical significance. Testing the distribution of data was done using one sample Kolmogorov-Smirnov test and accordingly parametric or non-parametric statistics is selected. Counts and percentage were used for describing and summarizing qualitative data, the arithmetic mean (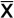) and the standard deviation (SD) were used as measures of central tendency and dispersion respectively for normally distributed quantitative data, the median was also used as a measure of central tendency for the non-normally distributed data. Mann-Whitney U test and Kruskal-Wallis H tests were done for comparing two or more independent quantitative non-normally distributed variables. Wilcoxon signed rank test was done for comparing two related quantitative non-normally distributed variables.

## Results

The present work was conducted on 40 mobile phones from patients and HCWs at AUSH. Ten mobile phones were randomly selected from each of 4 hospital departments: ICU, laboratory, dialysis unit and triage. 

This study enrolled the mobile phones of 16 (40%) nurses, 8 (20%) patients 7 (18%) workers, 5 (12%) laboratory technicians and 4 (10%) doctors. Half of these cell phones (50%) were touch screen phones and half (50%) were keypad phones. About 58% were new and 42% were old ones. The majority of these mobile phones did not have covers 27/40 (68%).

The current work revealed that the majority of isolated bacterial contaminants were mixed with more than one organism. It has been found that all mobile phones tested from the laboratory (100%) yielded mixed organisms, followed by 90% from dialysis unit and 70% from triage area. On the other hand, 60 % of the tested mobile phones from ICU revealed only one (single) isolate. The difference between these results was found to be highly statistically significant (p-value = 0.000). Regarding the categories of HCWs and patients, mixed bacterial contaminants had the upper hand in all categories. Of the 4 doctors tested mobile phones, 3 (75%) revealed more than one organism. The corresponding figures for nurses, lab technicians, workers and patients were as follows, 11/16 (69%), 5/5 (100%), 6/7 (86%), 5/8 (63%), respectively. This was found to be statistically significant (p-value = 0.040). In addition, of the 29 cell phones which were recorded to be cleaned by their owners, 21 (72%) yielded more than one organism. It has been also noted that the majority of individuals enrolled in the present study reported that they perform hand hygiene (HH) practices (37/40), of these 28 (76%) grew more than one organism from their cell phones. There was no statistical significant difference between any of these figures (p-value = 0.492) (Table 1 (Tab. 1)).

Observational surveys for HH compliance regarding World Health Organization (WHO) HH moment 1 (before touching the patient) and moment 4 (after touching the patient) [15] in the ICUs of the AUSH during the study period were as follows:

- Total HH compliance percentage for moments 1 and 4 in March 2014 = 378%. Nurses recorded the highest compliance rate (67%).
- Total HH compliance percentage for moments 1 and 4 in April 2014 = 42%. Nurses recorded the highest compliance rate (78%).

In this study, the bacterial count was performed by two methods; PP and SS. The mean bacterial count was found to be 357.10 CFU/ml, while the median was 13.00 CFU/ml by the PP method. The corresponding figures were 2192.03 and 171.50 organisms/phone using the SS method. SS method was found to yield much higher number of isolates than PP method in count categories of <10 (mean = 1294.9 and 4.5, respectively) and that of 10–<100 (mean = 1909.0 and 33.5, respectively). This was found to be statistically significant (p-value = 0.000). There was no statistical significant difference between the two methods regarding the high counts (100 or more) p=0.144 (Table 2 (Tab. 2)).

As regards isolated organisms in this study, methicillin-resistant *Staphylococcus aureus* (MRSA) was detected in 53% of the samples, followed by CoNS (50%), Bacillus (43%), Diphtheroids (30%), methicillin-susceptible *Staphylococcus aureus* (MSSA) (18%), *E. coli* and Viridans streptococci (13% each), Micrococci (10%), *Klebsiella pneumoniae* and ESBL *Klebsiella pneumoniae* (8% each). The least encountered isolates were *Acinetobacter baumanii* and Candida (3% each) (Table 3 (Tab. 3)).

In the present study, CoNS were the most frequently encountered isolates from doctors’ mobile phones (40%), followed by Bacillus spp. (20%), while MRSA, MSSA, diphtheroids and *E. coli* represented 10% each. On the other hand, MRSA was the most commonly isolated organism from nurses’ cell phones (20%), followed by Bacillus and CoNS (17% each). Regarding laboratory technicians, CoNS showed the highest percentage of isolation (26%), followed by Bacillus spp. and diphtheroids (21% each). MRSA has been isolated from 25% of workers’ mobile phones, while Bacillus accounted for 20% of isolates. As for patients, MRSA was the most frequently isolated organism (33%), followed by Viridans streptococci (27%) and CoNS (13%). Bacillus, micrococci and diphtheroids represented 7% each (Table 4 (Tab. 4)).

 MRSA were the most commonly encountered bacterial contaminants and were more frequently found in ICU (70%). Three ESBL *Klebsiella* spp. were isolated in the current study from ICU, laboratory and triage area. Two of the three isolates were revealed from workers mobile phones and one from a nurse cell phone. On the other hand, the one *Acinetobacter baumanii* strain encountered in this study was isolated from the mobile phone of a laboratory technician and was found to be multidrug-resistant. 

## Discussion

Hospital acquired infection caused by multidrug-resistant organisms is a growing problem in many health care institutions [16], [17], [18]. Hands, instruments, mobile phones or other inanimate hospital objects used by HCWs may serve as vectors for the nosocomial transmission of microorganisms [4], [19], [20], [21]. Unlike fixed phones, mobile phones are often used in these areas close to the patients and these patients are more vulnerable to hospital acquired infections [22], [23].

 In this study, 40 mobile phones from 4 different departments of a health care setting were screened for the presence of bacterial contamination. All of them were having one or more organism. Similar results were reported by Tagoe et al. (2011) [2] where all 100 mobile phones sampled were contaminated with varied numbers of bacteria. Ustun and Cihangiroglu (2012) [10] found that 98% culture-positive specimens were isolated from examined mobile phones of the HCWs. In a study conducted by Tambe and Pai (2012) [24] 83% of screened mobile phones of the HCWs showed bacterial or/and fungal contamination. 

In a separate study, researchers found that 95% of phones were contaminated with some kind of bacteria, many of which were resistant to multiple antibiotics. By also testing the participants’ hands, the researchers were able to show that a significant number of germs were transferred from their hands to their phones, and vice versa. In fact, about 30% of the bacteria on the phones ended up on the owner’s hands [25]. In a study done by Meadow et al. (2014) [26] they characterized microbial communities on smart phone touch screens to determine whether there was significant overlap with the skin microbiome sampled directly from their owners. They found that about 22% of the bacterial taxa on participants’ fingers were also present on their own phones. Beckstrom et al. (2013) [27] in their study of bacterial contamination of the parent’s cell phone in the NICU and the effectiveness of an anti-microbial gel in reducing transmission to the hands found that all cell phones demonstrated bacterial contamination. 90% had the same bacteria on the cell phone and their cleaned hands and 22% had no growth on their hands after applying anti-microbial gel after they had the same bacteria on the cell phone and hands.

Effective HH compliance in hospitals plays a key role in improving patient and provider safety, and in preventing the spread of HAIs. Despite this fact, HH compliance among HCWs in general is unacceptably low especially in developing countries [28]. During our study period, HH compliance rates among HCWs were estimated to be 37% and 42%. This was in concordance with the results of previous studies, where HH compliance rates ranged from 5%–89%; average, 39% [29]. In addition, in our study HH compliance was found to be higher among nurses (67% and 78%, respectively). This agreed with the findings of Rosenthal et al. in 2005 [30] and in 2013 [31]; where compliance was higher among nurses than among other HCWs.

 Lower rates of contamination were found by Kokate et al. (2012) [5] and Mark et al. (2014) [32] where both reported 60% contamination rates of examined mobile phones of HCWs. 

In the present study, the bacterial count was performed by two methods; PP and SS. It has been found that in low and moderate counts (<10 and 10 and more), SS method yielded statistically significant higher numbers of organisms than PP method, while in high counts (100 or more), though SS method revealed higher numbers of isolates than those yielded by PP method, yet this was not found to be statistically significant. SS method is also easier and less laborious compared to PP method. It has been used by many researchers for enumeration and detection of bacterial agents [10], [21], [24].

In the study by Pal et al. (2013) [33] the median colony count for touch screen phones and keypad devices was 0.09 CFU/cm^2^ (interquartile range (IQR) 0.05–0.14) and 0.77 CFU/cm^2^ (IQR range 0.45–3.52), respectively. Results from the study of Tagoe et al. (2011) [2] showed high levels of bacterial contamination of mobile phones used by students in the University of Cape Coast with an overall mean viable bacterial count of 9.9×10^5^ CFU/phone using PP method. A United Kingdom study tested 30 mobile phones for levels of potentially harmful bacteria or the total viable bacterial count (TVC). The results revealed that 25% exceeded the acceptable TVC by 10 times and have 18 times the TVC as a handle on a public restroom toilet [34]. It was estimated that the average cell phone harbors 25,107 bacteria per square inch [25]. In general; the greater the concentration of the microbe, the longer it survives and survival can range from minutes to months. This is a cause for concern since these pathogenic isolates are capable of causing diseases in anyone who gets contaminated whilst using the mobile phone [2]. 

 In the current study, MRSA was detected in 53% of the samples, followed by CoNS (50%), Bacillus (43%), diphtheroids (30%), MSSA (185%), *E. coli* and Viridans streptococci (13% each), micrococci (10%), *Klebsiella pneumoniae* and ESBL *Klebsiella pneumoniae* (8% each) and finally *Acinetobacter baumanii* and Candida (3% each). A nearly similar result was reported by Angadi et al. (2014) where MRSA was isolated from 53.3% of HCWs mobile phones [7]. In the study by Tagoe et al. (2011) [2] the isolated bacteria included *Klebsiella pneumoniae* (10%), Citrobacter spp. (2%), *S. aureus* (4%), CoNS (15%), *Pseudomonas aeruginosa* (4%), *Salmonella* spp. (3%), *Shigella* spp. (2%), *Proteus mirabilis* (19%), *E. coli* (8%), *Bacillus cereus* (23%), *Streptococcus pneumoniae* (10%), *Salmonella* spp. (3%) and *Shigella* spp. (2%).

Tambe and Pai (2012) [24] reported that the isolation of *S. aureus* was maximum in all the categories of HCWs (54%), followed by micrococci (21%), diphtheroids (8%), enterococci (4%), Pseudomonas, Citrobacter and *Bacillus* spp. (3% each), Acinetobacter, Enterobacter and *Streptococcus viridans* (2% each). In the study by Kokate et al. (2012) [5] CoNS was the dominant organism (72%) followed by diphtheroids (22%) and *Aspergillus niger* 2 (6%). Rana et al. (2013) [1] found that out of the 50 samples from HCWs, 10 were contaminated with *S. aureus*, 4 CoNS, one *E. coli* and *Pseudomonas* spp. together. Of the 10 *S. aureus* 40% were resistant to methicillin. 

In a study by Bhoonderowa et al. (2014) [35] CoNS was the most prevalent (69 %) bacteria from mobile phones of volunteers in the community. In 2014, a study carried out by Raghavendra et al. [36] revealed that 52% of the examined mobile phones of HCWs were contaminated by *S. aureus*. In this work, it has been noted that staphylococci were the most frequently encountered isolates. This pathogen is of greater concern because of its virulence, its ability to cause a diverse array of life threatening infections, and its capacity to adapt to different environmental conditions [37]. It is also a well known fact that organisms like *S. aureus* and CoNS resist dryness and thus can survive and multiply rapidly in warm environments like cell phones [38]. 

In a study carried out by Ustun and Cihangiroglu (2012) [10] MRSA and ESBL-producing *E. coli* were detected in 10% and 11% of mobile phones samples, respectively. Pal et al. [33] reported that 13% of phones grew either MRSA or vancomycin-resistant enterococci. 

The observed high rate of antibiotic-resistant bacteria (MRSA and ESBL *Klebsiella pneumoniae*, accounting for 60% of the isolates) in this study could be attributed to both the misuse and abuse of antibiotics. The prevalence of antibiotic-resistant bacteria is a serious problem with important implications for hospital infection prevention and control program. Although the geographic distribution of these bacteria is worldwide, the epidemiology and dissemination patterns appear to differ within and across regions [33].

In the present study, there was no statistical significant difference in the mean viable bacterial count isolated from different departments of the hospital or among different categories of HCWs or with the cleanliness of mobile phones or implementation of hand hygiene practices. However, mixed infection with more than one type of organism had the upper hand in this study. It was more frequently observed in samples obtained from the hospital laboratory (100%), followed by those from dialysis units (90%) and triage (70%). On the other hand, 60 % of the tested mobile phones from ICU revealed only one isolate. Srikanth et al. (2010) [39] reported that polymicrobial growth was detected in 71% of HCW mobile phones and 78% of corporate mobile phones. In addition, Chawala et al. [40] documented that the majority of HCWS mobile phones showed polymicrobial growth i.e. 40% mobile phones showed two types of organisms, 28% showed the presence of three or more types of organisms and only 25% were mono microbial. On the contrary, in the study conducted by Ulger et al. (2009) [41], it was found that 49% of phones grew only one bacterial species, 34% two different species, and 12% three or more different species.

At the same time, mixed infection was found more among laboratory technicians followed by workers than among doctors and nurses. Technicians in the hospital laboratory are often exposed to a wide range of pathogenic and multi-resistant micro-organisms during handling different types of samples in their work. In the study conducted by Tambe and Pai (2012) [24] the isolation of bacterial flora was seen to a greater extent among the laboratory technicians and the ward boys as compared to the nurses and the doctors. Similar findings were reported by Trivedi et al. (2011) [38] as the highest bacterial contamination of mobile phones (52%) were found among HCWs other than nurses and doctors, followed by nurses (50%) and finally doctors (38%).

 A practice guideline was issued by the community and Hospital Infection Control Association (CHICA, Canada) to address the issues of electronic devices in health care settings. Some of their recommendations include that hand hygiene should be performed between patient contact and before and after accessing a device, manufacturer’s guidelines for use, cleaning/disinfection and maintenance should be reviewed to ensure that these guidelines meet the standards for cleaning and low-level disinfection that are necessary for exposure to multidrug-resistant organisms [42]. 

## Conclusions

- Mobile phones were found to be highly contaminated with bacterial agents.
- Their usage in hospital settings serves as a potential vehicle for the spread of nosocomial pathogens including multidrug-resistant pathogens as MRSA.
- The surface spread method is a simple and useful tool for detection and enumeration of bacterial agents contaminating mobile phones.

### Recommendations

- Screening of mobile phones for bacterial contamination is recommended especially within hospital critical areas.
- Due care should be taken when using mobile phones in health care settings especially during working hours to reduce the risk of transmission of detrimental bacterial agents.

## Notes

### Competing interests

The authors declare that they have no competing interests relevant to this paper.

### Acknowledgment

We would like to express our deep thanks and sincere appreciation to Dr. Ashraf Wahdan, lecturer of biostatistics at the High Institute of Public Health, for his kind effort in the statistical analysis performed in this study. We would also like to extend our hearty thanks and deepest gratitude to the members of the infection control team at the Alexandria University Students’ Hospital.

## Figures and Tables

**Table 1:**
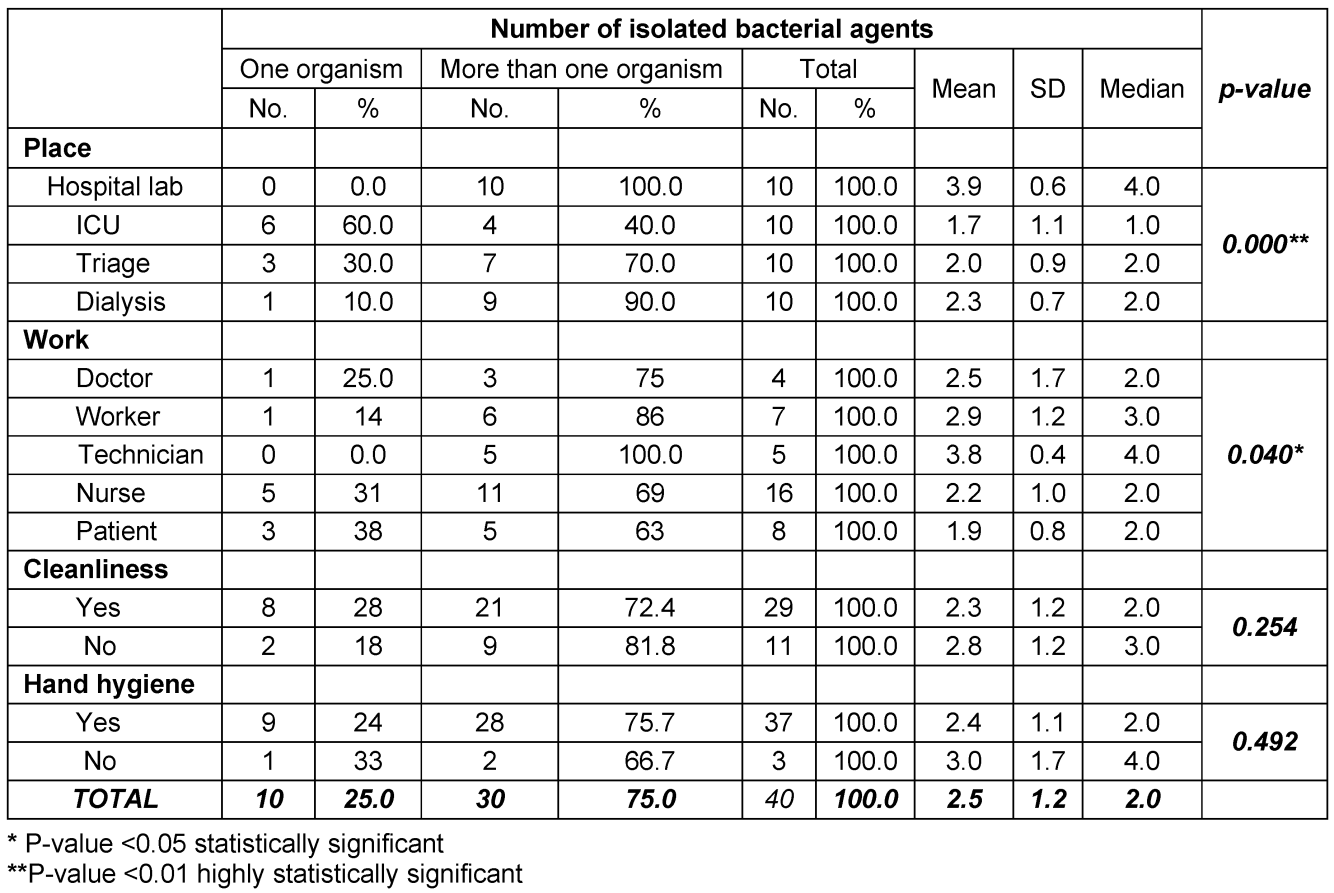
Number of isolated bacterial agents in relation to place, work, mobile cleanliness and hand hygiene practices

**Table 2:**
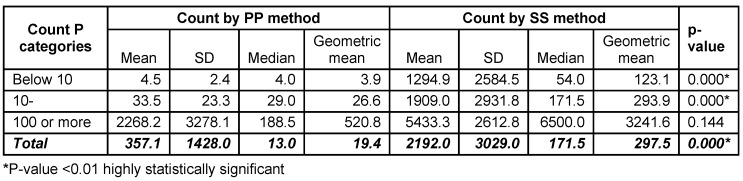
Count of bacterial agents contaminating the 40 tested mobile phones using pour plate and surface spread methods

**Table 3:**
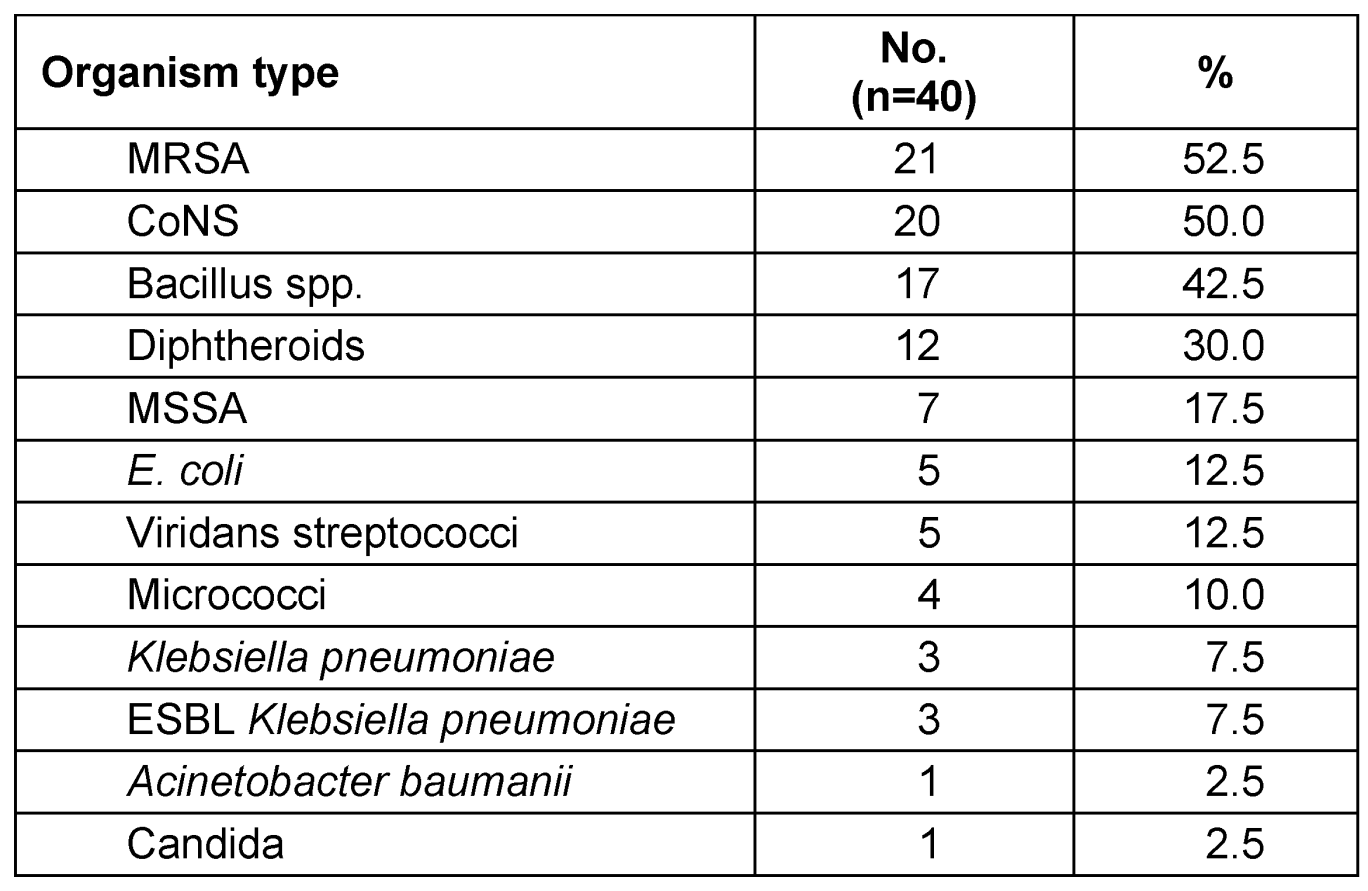
Types of isolates from the 40 tested mobile phones

**Table 4:**
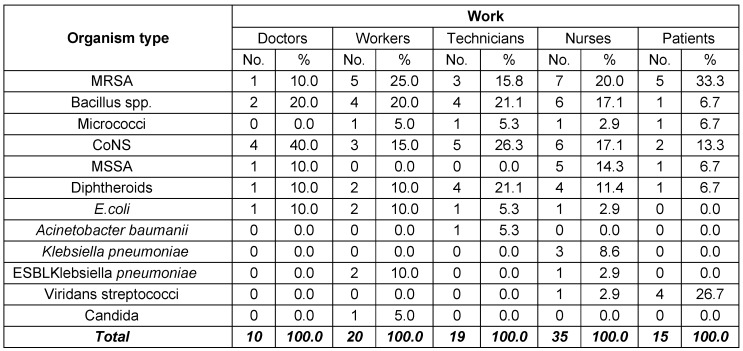
Distribution of isolates according to owners of 40 tested mobile phones
